# The effect of obesity on spirometry tests among healthy non-smoking adults

**DOI:** 10.1186/1471-2466-12-10

**Published:** 2012-03-21

**Authors:** Mohammed Al Ghobain

**Affiliations:** 1 Department of Medicine, College of Medicine, King Saud bin Abdulaziz University for Health Sciences, Riyadh, P.O. Box 90068 11321, Saudi Arabia

**Keywords:** obesity, spirometry, Saudi Arabia

## Abstract

**Introduction:**

The effects of obesity on pulmonary functions have not been addressed previously among Saudi population. We aim to study the effects of obesity on spirometry tests among healthy non-smoking adults.

**Methods:**

A cross sectional study conducted among volunteers healthy non-smoking adults Subjects. We divided the subjects into two groups according to their BMI. The first group consisted of non-obese subjects with BMI of 18 to 24.9 kg/m2 and the second group consisted of obese subjects with BMI of 30 kg/m2 and above. Subjects underwent spirometry tests according to American thoracic society standards with measurement of the following values: the forced vital capacity (FVC), forced expiratory volume in one second (FEV1), peak expiratory flow rate (PEF) and forced mid-expiratory flow (FEF25-75).

**Results:**

The total subjects were 294 with a mean age of 32 years. There were 178 males and 116 females subjects. We found no significant differences in FEV1 (p value = 0.686), FVC (p value = 0.733), FEV1/FVC Ratio (p value = 0.197) and FEF25-75 (p value = 0.693) between the obese and non-obese subjects. However, there was significantly difference in PEF between the two groups (p value < 0.020).

**Conclusion:**

Obesity does not have effect on the spirometry tests (except PEF) among health non-smoking adults. We recommend searching for alternative diagnosis in case of findings abnormal spirometry tests results among obese subjects.

## Introduction

Obesity is a chronic medical condition characterized by an excessive accumulation of fat on human body that causes a generalized increase in body mass. It is measured by using body mass index (BMI) which is a reflection of weight and height. Body mass index (BMI) is calculated as the weight in kilograms divided by the square of the height in meters (BMI = weight (kg)/height (m2)). The world Health Organization (WHO) classified obesity using BMI cut-off values of 25 and 30 kg/m2. Body mass index (BMI) of 18 to 24.9 kg/m2 is considered normal weight, a BMI of 25.0-29.9 kg/m2 is considered overweight and a BMI of 30 kg/m2 or higher is considered obesity [1].

Obesity has been associated with many health consequences, including but not limited to diabetes, hypertension, hyperlpidemia, ischemic heart diseases, obstructive sleep apnea, stroke, premature death, osteoporosis and a reduction of the overall quality of life [2]. Obesity affects the respiratory system as well. To investigate the effect of obesity on the respiratory system, most researchers use values of pulmonary function tests (PFT). The factors that usually affect the values of pulmonary function tests are age, gender, height, race or ethnic origin and possibly obesity. As the individual gets older age, the lung volumes and capacities become smaller and the lung volumes and capacities are larger in males than females [3]. A short man will have generally a smaller PFT results than a taller man of the same age. Whites tend to have slightly larger trunks and shorter legs compared to Blacks at a given height which corresponds to vital capacities that are 10-15% larger for a given standing height [4,5] Blacks, Hispanics and Native Americans have different PFT results compared to Caucasians. Ethnic differences in PFT have also been suggested for many other groups specifically Asians [6] Latin Americans [7] Indians [8] and South Africans [9].

Weight may have effects on pulmonary function tests including impairment on pulmonary function testing, small airway dysfunction and expiratory flow limitation, alterations in respiratory mechanics, decreased chest wall and lung compliance, decreased respiratory muscle strength and endurance, decreased pulmonary gas exchange, lower control of breathing, and limitations in exercise capacity [10-13].

The interaction between obesity and PFT has not been addressed previously among the Saudi Arabian population. Therefore, we aim to study the effects of obesity on spirometry tests among healthy non-smoking adults in Saudi Arabia. Our study is limited to spirometry part of PFT because spirometry tests are considered to be the initial screening tool for pulmonary diseases, they are the most widely used tests, and they are easy to conduct using equipment that is available in all pulmonary functions laboratories.

## Methods

This is a cross sectional study conducted at the pulmonary function laboratory of king Abdulaziz medical city, Riyadh, Saudi Arabia. The subjects were selected from the adult male and female population (aged 18 to 75 years) of healthy volunteers or hospital visitors or relatives of patients visiting the hospital. The subjects who accepted the invitation underwent a medical evaluation including a meticulous and thorough medical history, and a full physical examination. Subjects were Saudis lifetime nonsmoker for either cigarettes or water pipes (Shisha) with a weight ranges from 40 kg to 120 kg and height range from 140 cm to 190 cm. They had no history of any respiratory complaints like cough, shortness of breath, wheezes or fever or history of upper respiratory tract infection in the past 4 weeks or history of respiratory diseases such as pulmonary tuberculosis or asthma and not having history of cardiac or thoracic surgery or features suggestive of cardiac or lung disease or evidence of chest deformities or serious medical conditions, and not having worked in environments with a high concentration of dust or pollution.

The sex, age, standing height and body weight of all subject met the inclusion were recorded. The weight was measured with the subjects wearing light clothing and barefoot on a SECA weighting scale (Hamburg, Germany). The standing height was measured without shoes with the subject's back to a vertical backboard. Both the heels were placed together, touching the base of the vertical board. Normal weight and obesity were defined on the basis of WHO cutoffs. The spirometry tests were conducted using a Micro-Loop Viasys Healthcare (Ireland). The spirometry device was calibrated with JAEGER calibration pump using 3.0 L syringe at three flow rates, in accordance with the manufacturer's recommendations, before each day's testing and after every few hours of testing. All Subjects underwent spirometry tests, using techniques recommended by the American Thoracic Society (ATS). The spirometry test was done in the morning by at least two trained qualified technicians. The subjects underwent the spirometry test in the sitting position, wearing a nose clip. Uniformity of spirometry test was assured by using the same device brand for all the subjects. The validity of the test was verified according to the ATS recommendations. The spirometry tests measured were the forced vital capacity (FVC), forced expiratory volume in one second (FEV1), peak expiratory flow rate (PEFR) and forced mid-expiratory flow (FEF25-75%). In addition to these measured parameters, the ratio of FEV1 to FVC (FEV1/FVC, expressed as a percentage) was calculated. The subjects were divided into two groups according to their BMI. The first group consisted of non-obese (normal body weight) subjects with BMI of 18 to 24.9 kg/m2 and the second group consisted of obese subjects with BMI of 30 kg/m2 and above

The statistical analysis was performed using the SPSS 18 software (SPSS, Chicago). Descriptive statistics were calculated for the total study sample, for males and females and for both groups using means and standard deviations. The variables were expressed as the means and standard deviations, and *p *value less than 5% was considered statistically significant. Independent samples test was used to compare the spirometry results of obese to non-obese subjects. The Institutional Review Board at King Abdullah International Medical Research Centre (KAIMRC) approved the study and written informed consent was obtained from all subjects.

## Results

The baseline characteristics of study subjects are shown in Table 1. The total study population was 294 subjects with a mean age of 32 years. There were 178 males subjects (60.5%) and 116 females subjects (39.5%). We found no significant difference in age (p value = 0.974) or BMI (P value = 0.755) between the males and females subjects. With regard to spirometry tests, we found significant differences in the FEV1 (p value < 0.001), FVC (p value < 0.001), PEF (p value < 0.001) and FEF 25-75 (p value < 0.004), between the male and female subjects (Table 1). We grouped the data from males and females together because there was no significant difference between males and females for the effects of BMI on the spirometric values (p value = 0.423).

**Table 1:** The baseline characteristics and spirometry tests variables of the study subjects

	*Male (n = 178)*		*Females (n = 116)*			*Total (n = 294)*	
	
	*Mean*	*Std. Dev*	*Mean*	*Std. Dev*	*P value*	*Mean*	*Std. Dev*
Age (years)	*32.19*	10.164	*32.15*	*9.639*	*.974*	32.17	9.943
Height (cm)	*171.87*	6.261	*159.16*	*5.975*	*< .001*	166.85	8.739
Weight (kg)	*84.21*	18.212	*71.50*	*18.325*	*< .001*	79.19	19.258
BMI (kg/m^2^)	*28.503*	5.9454	*28.253*	*7.1559*	*.755*	28.404	6.4395
FEV1 (L)	*3.6363*	.44796	*2.6692*	*.37764*	*< .001*	3.2548	.63356
FEV1%	*92.51*	9.938	*93.48*	*9.554*	*.406*	92.89	9.784
FVC (L)	*4.4958*	.56783	*3.2129*	*.47231*	*< .001*	3.9897	.82270
FVC %	*95.83*	10.109	*97.99*	*10.522*	*.078*	96.68	10.311
PEF(L/min)	*560.47*	76.552	*393.54*	*51.952*	*< .001*	494.61	106.201
PEF %	*100.76*	13.228	*98.80*	*12.942*	*.211*	99.99	13.129
FEV1/FVC Ratio	*80.98*	3.646	*82.72*	*2.954*	*< .001*	81.67	3.489
FEF 25-75 (L)	*3.4693*	.71140	*2.6894*	*.48920*	*< .001*	3.1616	.73854
FEF 25-75%	*75.59*	15.894	*70.95*	*11.262*	*.004*	73.76	14.406

In comparing the spirometric values between non-obese and obese subjects, we found no significant differences in FEV1 (p value = 0.686), FVC (p value = 0.733), FEV1/FVC Ratio (p value = 0.197) and FEF25-75 (p value = 0.693) between the two groups. However, there was significantly difference in PEF between the two groups (p value < 0.020). The obese subjects had lower PEF values than those non-obese subjects (Table 2).

**Table 2:** Independent sample test comparing age, BMI and spirometric variables between non-obese and obese subjects

	Group 1 (non-obese)		Group 2 (obese)				
	**Mean**	**SD**	**Mean**	**SD**	**Mean difference**	**95% CI**	**P value**

**Height (cm)**	166.75	8.55	166.95	8.93	-0.19	(-2.20, 1.81)	0.848

**Weight (kg)**	62.40	8.36	94.67	12.16	-32.26	(-34.67, -29.84)	< .00001

**BMI (kg/m^2^)**	22.37	1.82	33.95	3.47	-11.58	(-12.22, 10.93)	< .00001

**FEV1 (L)**	3.27	0.56	3.24	0.69	0.02	(-0.11, 0.17)	0.686

**FEV1%**	91.95	9.41	93.76	10.06	-1.81	(-4.05, 0.42)	0.110

**FVC (L)**	4.00	0.75	3.97	0.88	0.03	(-0.15, 0.22)	0.733

**FVC %**	95.83	10.10	97.46	10.47	-1.63	(-4.00, 0.73)	0.175

**PEF(L/min)**	508.42	113.65	479.62	95.62	-28.80	(-53.02, -4.59)	0.020

**PEF %**	103.09	13.13	96.62	12.30	-6.46	(-9.39, -3.53)	< .00001

**FEV1/FVC Ratio**	81.94	3.86	81.41	3.09	0.53	(-0.26, 1.33)	0.197

**FEF 25-75 (L)**	3.14	0.63	3.17	0.82	-0.03	(-0.20, 0.13)	0.693

**FEF 25-75%**	72.12	13.01	75.27	15.47	-3.14	(-6.44, 0.14)	0.061

## Discussion

The main results of our study showed that there were no significant differences between the obese and non-obese subjects in FEV1, FVC, FEV1/FVC ratio and FEF 25-75; however, there was a significant difference between the two groups in regard to PEF. The obese subjects had lower PEF values than the non-obese subjects. Low PEF in obese subjects can be explained by an increase in total respiratory resistance and airway resistance with obesity. The higher airway resistance, the higher BMI and subsequently, the lower PEF [14].

To our knowledge, this is the first study to address the relationship between obesity and spirometry tests among the Middle East population. Many studies conducted in the rest of the world and addressed such relationship showed heterogeneous results. The effects of obesity on spirometric values are not consistent across all studies with some studies shown no effects and some other studies shown positive effects. This discrepancy between studies could be explained by the wide variations in ethnicity of different population in PFT values or this may be a result of methodological differences in these studies.

However all studies that addressed other pulmonary function tests values (e.g. lung volumes and capacities) showed that the obesity directly correlated with these values. Our study was limited to the spirometric values (FEV1, FVC, FEV1/FVC, FEF 25-75 and PEF) and did not include the other pulmonary function tests (e.g. lung volumes and capacities).

In contrast to other studies, a study conducted by Costa D et al. addressed the relationship between spirometric tests and obesity produced results similar to those of our study. The author recruited 20 obese young women with a BMI of 35-49.99 kg/m2 who were sedentary, non-smokers and had no lung disease and 20 non-obese control young women who were also sedentary non-smokers and had no lung diseases with body mass indices between 18.5 and 24.99 kg/m2. There were no significant differences between the obese group and the non-obese group with respect to the age, vital capacity (VC), tidal volume (TV), FVC, and FEV1. However, the obese group had a greater inspiratory reserve volume (IRV), a lower expiratory reserve volume (ERV), and lower maximal voluntary ventilation (MVV) than the non-obese group [15].

Jones RL et al. studied pulmonary function test results from 373 patients with wide range of BMIs. He found significant inverse relationships between BMI and the values of VC and total lung capacity (TLC). Moreover, the functional residual capacities (FRC) and ERV decreased exponentially with increasing BMI, to the extent that morbid obesity resulted in the patients breathing near their residual volumes (RV) [16]. According to Koenig, this fact is attributable to pressing the diaphragm upwards due to the expanded abdominal volume of obese individuals [11].

In mild obesity, the spirometry results may be normal or may suggest a restrictive process, with a symmetric reduction in FEV1 and FVC [17] on other hand, The VC and TLC are well preserved in mild obesity because there is a compensatory increase in inspiratory capacity (IC). It is therefore important to be able to separate the changes that may be explained by an increased BMI from those that may be related to another process. At present, there is no way to differentiate these factors other than a conducting a careful history and physical examination combined with other clinical data [18].

The most important change in pulmonary functions in obesity is a decrease in lung compliance due to the increased the weight of chest wall and the higher position of diaphragm in the thoracic cavity resulting in a decrease in the lung functions which subsequently leads to increase in work of breathing [16]. In addition, the deposition of fat on the chest wall may impede the expansion and excursion of the rib cage, through a direct loading effect or by altering the inter-costal muscle function [19]. Furthermore, obesity has been shown to be associated with markers of systemic and vascular inflammation such as the hormone leptin [20]. These inflammatory factors may exert local effects on lung tissue, leading to subtle reductions in airway diameter.

The Strength of our study is the recruitment of subjects who were healthy without co-morbidity and the selection of subjects for the study who had been seen by a physician prior to being tested, and there were no indications that they had any co-morbidity. Anther strength of this study is that it is the first study in Saudi Arabia and among Middle East population to address the relationship between obesity and spirometry tests values.

Our study had the limitation of using BMI as an indictor of obesity. BMI is a global measure of body mass that includes both fat and lean mass and takes no account of differences in fat distribution. If the reduction of lung volumes in obesity is due to a direct mechanical effect on lung volumes, then the distribution of body fat should modify the relationship between BMI and lung volumes. Abdominal and thoracic fat are likely to have direct effects on the downward movement of the diaphragm and on chest wall properties, while fat on the hips and thighs would be less likely to have any direct mechanical effect on the lungs. However, the standard classification of obesity uses BMI as a reflection of obesity and this classification is used globally by WHO and other related health organizations. It is considered to be the gold standard at the present time.

Another limitation in our study is that we did not measure all the pulmonary function tests variables; our study was limited to the spirometry tests values and did not include lung volumes measurements. We elected to limit our study to the spirometry tests values because these values had not been previously investigated in relation to obesity in the Saudi population, and will be very difficult to apply data from other parts of the world to the Saudi population due to the differences in ethnicity. Our study addressed an important gap in the literature regarding the effects of obesity on spirometry tests values among Saudi population.

In conclusion, obesity does not have direct effect on the spirometry tests results (except PEF) among health non-smoking adults Saudis and if there is any effect, it should be explained by alternative diagnosis or underlying respiratory diseases. We strongly recommend conducting larger study including all pulmonary functions tests variables (spirometry tests and other lung volumes) among Saudi population, moreover, it will interesting to use other indices of obesity like abdominal girth, sub-scapular skin fold thickness, and the ratio of abdominal girth to hip breadth as reflection of obesity instead of BMI and find if such results will differ in comparison to the results of using BMI.

Based on our result, we highly recommend to physicians who use spirometry test in their practice to search for alternative diagnosis in case of findings abnormal spirometry tests results among obese individuals as these abnormal findings should not attributed to obesity per se.

## Conflict of interests and support

The authors declare that they have no competing interests.

## Authors' contributions

MG, primary author, study design, study conducting, analysis, writing and reviewing the manuscript.

## Pre-publication history

The pre-publication history for this paper can be accessed here:

http://www.biomedcentral.com/1471-2466/12/10/prepub
